# Minimal cross-trial generalization in learning the representation of an odor-guided choice task

**DOI:** 10.1371/journal.pcbi.1009897

**Published:** 2022-03-25

**Authors:** Mingyu Song, Yuji K. Takahashi, Amanda C. Burton, Matthew R. Roesch, Geoffrey Schoenbaum, Yael Niv, Angela J. Langdon

**Affiliations:** 1 Princeton Neuroscience Institute, Princeton University, Princeton, New Jersey, United States of America; 2 National Institute on Drug Abuse Intramural Research Program, NIH, Baltimore, Maryland, United States of America; 3 Department of Psychology, University of Maryland, College Park, Maryland, United States of America; 4 Program in Neuroscience and Cognitive Science, University of Maryland, College Park, Maryland, United States of America; 5 Department of Psychology, Princeton University, Princeton, New Jersey, United States of America; Ghent University, BELGIUM

## Abstract

There is no single way to represent a task. Indeed, despite experiencing the same task events and contingencies, different subjects may form distinct task representations. As experimenters, we often assume that subjects represent the task as we envision it. However, such a representation cannot be taken for granted, especially in animal experiments where we cannot deliver explicit instruction regarding the structure of the task. Here, we tested how rats represent an odor-guided choice task in which two odor cues indicated which of two responses would lead to reward, whereas a third odor indicated free choice among the two responses. A parsimonious task representation would allow animals to learn from the forced trials what is the better option to choose in the free-choice trials. However, animals may not necessarily generalize across odors in this way. We fit reinforcement-learning models that use different task representations to trial-by-trial choice behavior of individual rats performing this task, and quantified the degree to which each animal used the more parsimonious representation, generalizing across trial types. Model comparison revealed that most rats did not acquire this representation despite extensive experience. Our results demonstrate the importance of formally testing possible task representations that can afford the observed behavior, rather than assuming that animals’ task representations abide by the generative task structure that governs the experimental design.

## Author summary

To study how animals learn and make decisions, scientists design experiments, train animals to perform them, and observe how they behave. During this process, an important but rarely asked question is how animals understand the experiment. Merely through observing animals’ behavior in a task, it is often hard to determine if they understand the task in the same way as the experimenter expects. Assuming that animals represent tasks differently than they actually do may lead to incorrect interpretations of behavioral or neural results. Here, we compared different possible representations for a simple reward-learning task in terms of how well these alternative models explain animal’s choice behavior. We found that rats did not represent the task in the most parsimonious way, thereby failing to learn from forced-choice trials what rewards are available on free-choice trials, despite extensive training on the task. These results caution against simply assuming that animals’ understanding of a task corresponds to the way the task was designed.

## Introduction

Much knowledge of the world is acquired not from instructions, but through observations and inference. For example, you might choose which campus cafeteria to visit by checking their daily menus. Eventually, you may realize that cafeterias A and B have the same menu (unbeknownst to you, they are run by the same caterer). This implicit knowledge allows you to apply whatever you learn about one dining location to the other: upon hearing that cafeteria A is serving your favorite dish, you can get it at the close-by cafeteria B.

Acquiring such knowledge can be considered as learning the structure of a task, or in reinforcement learning (RL) terminology, learning a state representation for the task [1–3]. A state representation forms the basis upon which values (expectations about future rewards) and policies (rules for action in different settings) can be learned [4]. In tasks in which different settings (e.g., cafeteria A or B) lead to the same outcome (the same dishes on the menu), the state representation for A and B can be *shared*. The benefit of this is two-fold: first, it allows compression of state representations, excluding irrelevant variation and thus reducing the complexity of the learning problem. Second, it accelerates learning as only a single experience of a tasty salad in cafeteria A is required in order to exploit that knowledge in both locations. While a range of alternative state representations can support learning in any given setting, one that matches the “true” underlying structure of a task supports efficient learning and accurate task performance.

The challenge for a learner to build an appropriate state representation is particularly acute when there is no explicit instruction on the “rules” for solving a task. This occurs by necessity in experiments on non-human animals, in which subjects are trained solely through ongoing experience. Experimenters know the ground-truth structure of a task, and often assume the subjects understand it similarly. However, even relatively simple tasks may be represented in a multitude of ways, often with only subtle differences in overt behavioral performance. Despite rapid progress in the development of artificial learning algorithms that can extract appropriately abstract task representations from reinforcement [5–8], it remains unknown how animals form a state representation solely through their experience of stimuli, rewards and the contingency of each of these on their actions. In particular, it is an open question how animals might generalize their learning about upcoming rewards across distinct features of experience, thereby building a concise state representation of a task.

## Results

**Fig 1:**
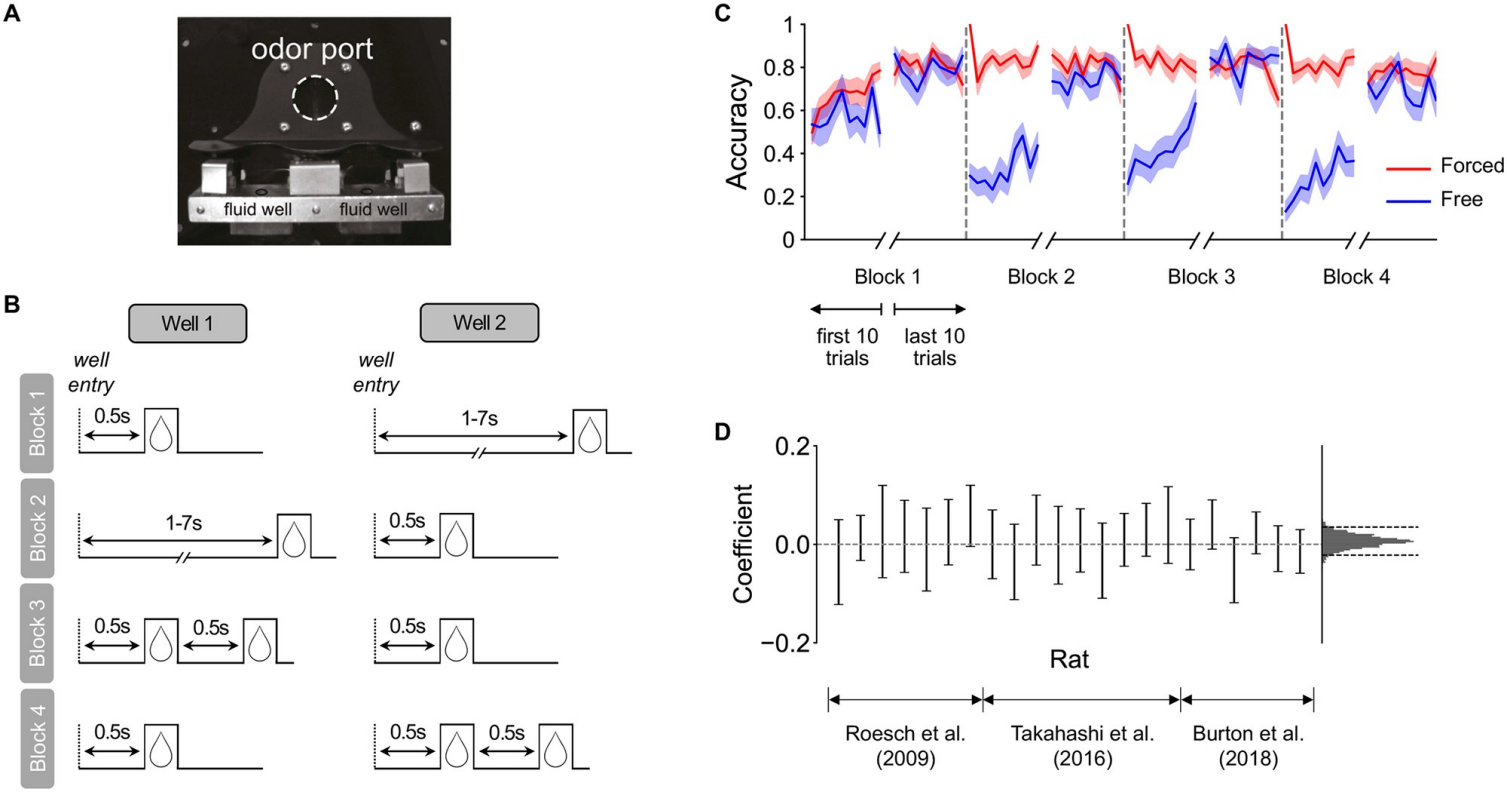
The odor-guided choice task and animals’ behavior. **(A)** The experiment apparatus included an odor port and two fluid wells where rewards were delivered. To start each trial, the animal first poked into the odor port; after 0.5 seconds, one of three odors was delivered, signaling the current trial type. One odor signaled a left forced-choice trial, another a right forced-choice trial, and a third indicated free choice between left and right wells. After odor offset, the animal could make a choice by entering either the left or right fluid wells. Reward was delivered if they made the correct choice on a forced-choice trial and as long as they successfully made one of the choices on a free-choice trial. **(B)** Block sequence in an example session. Sessions always started with two “delay blocks” (blocks 1 and 2), followed by two “magnitude blocks” (blocks 3 and 4). In block 1, the “short” reward (delivered 0.5s after well entry) was available in one well and the “long” reward (delivered 1–7s after well entry) in the other; the reward contingency switched between the wells on block 2. In block 3, “long” reward then changed to “big” reward (two sucrose drops delivered 0.5s after well entry), while “short” reward stayed the same but is now referred to as “small” reward (one sucrose drop) in comparison to the alternative; these reward contingencies were switched again on block 4. The well that was initiated with the better (short) reward option was randomized across sessions. **(C)** Learning curves for forced-choice (red) and free-choice (blue) trials. The curves are aligned to block-switch points (gray dashed lines), with the first and last 10 trials of each block shown. Accuracy is evaluated as the percentage of trials the animal chose the better option for that trial type (forced-choice trials: the rewarded well; free-choice trials: the well with reward at shorter delay or larger amount). Shaded areas represent 1 s.e.m across animals (*N* = 22). **(D)** Coefficients of a hierarchical logistic regression predicting the accuracy of the first free-choice trial after a previous incorrect free-choice as a function of the number of intervening correct forced-choice trials. Left (error bars): coefficients for individual animals, ordered by dataset, with error bars representing 95% highest posterior density interval (HDI). Right (histogram): the posterior distribution of the group mean, with dashed lines representing 95% HDI. At both individual and group levels, 95% HDI of the coefficients overlapped with zero, suggesting that there was minimal generalization of learning from correct forced-choice trials to subsequent free-choice trials.

We directly tested the extent of generalization in learned state representations that guide choice behavior in an odor-guided decision-making task in rats [9]. Rats were trained to sample an odor at a central odor port, before responding at one of two fluid wells (Fig 1A). The odor stimulus provided a cue for which of two wells would be baited with a sucrose reward. Two odors signalled “forced choice” trials, one indicating reward will be available in the left well, and one indicating the right well. In either case, choosing the unrewarded well terminated the trial immediately. A third odor—“free choice”—indicated reward will be available in either well. Importantly, if a “valid” well were chosen on any trial (i.e., the rewarded well on forced-choice trials, or either well on free-choice trials), the delay to and amount of reward was determined by the side of the well, not the odor. Unsignaled to the animal, in each block of the task, one well delivered a “better” reward outcome, either at a shorter delay or a larger amount than the other well; reward contingencies changed between blocks during a session (see Fig 1B for details).

Because of the shared reward setting across odors, it would be beneficial for the animal to acquire a representation of the task in which learning from valid forced-choice trials generalizes to the same well location in free-choice trials. This representation aligns with the underlying generative structure of the task, and would support faster learning when reward contingencies change between blocks.

To study how rats interpret the structure of this odor-guided choice task, we collated behavior from several experiments using the same behavioral paradigm [10–12]. On average, across sessions, rats learned to choose the well with the better reward on free-choice trials within each block, while maintaining high choice accuracy on forced-choice trials throughout the session (Fig 1C). To determine whether rats learned to choose the better option on free-choice trials by generalizing from rewards delivered on forced-choice trials (and not from experience in free-choice trials alone), we first performed a behavioral analysis. If animals had knowledge of the shared reward setting, reward outcomes in valid forced-choice trials should provide information about the reward available in the two wells, and as a result, improve performance on subsequent free-choice trials. We therefore conducted a hierarchical logistic regression predicting the accuracy of free-choice trials as a function of how many rewarded forced-choice trials the animal had experienced since the last *incorrect* free-choice trial (i.e., the last time they chose the worse well). A positive coefficient would indicate use of forced-choice experience to inform free-choice decisions. We did not find evidence for such signature of generalization (Fig 1D), either at the group level (95% highest posterior density interval (HDI) of the group mean of the coefficient: [-0.022, 0.035]), or for individual animals (95% HDI of individual coefficients all include zero). Adding trial index in the block as an additional regressor (to account for the increase in accuracy over each block) did not change the results.

**Fig 2:**
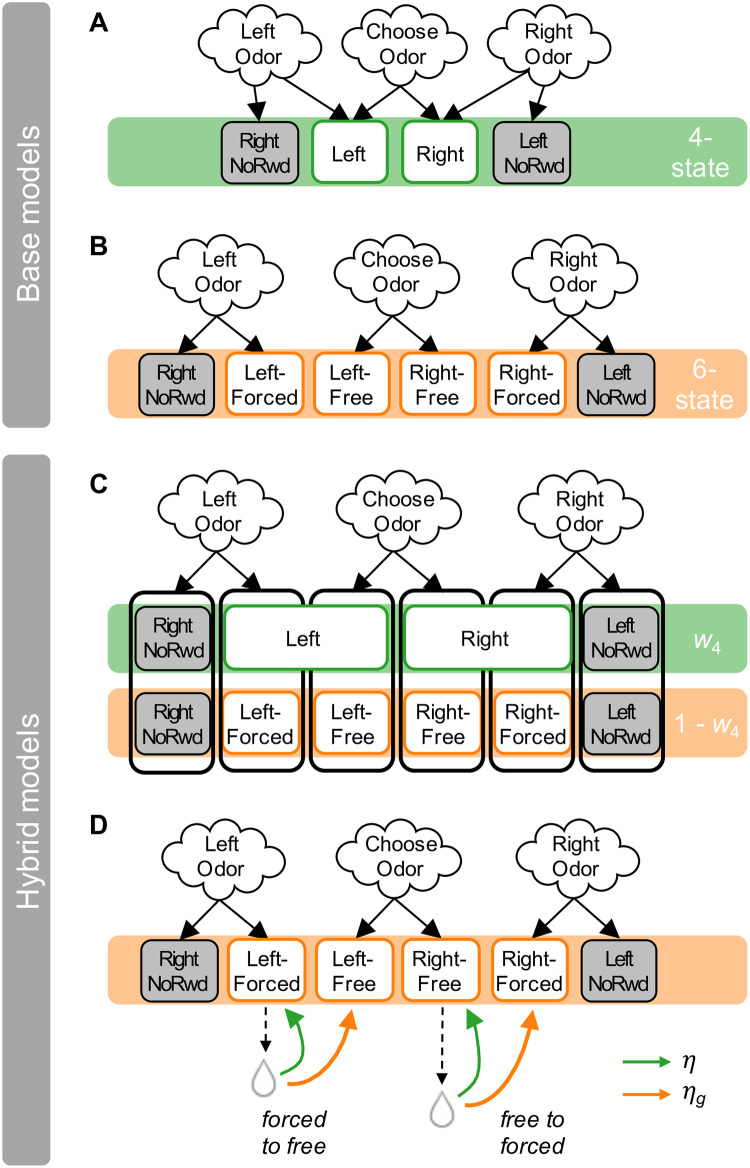
State representations of RL models. **(A) The four-state model**: free-choice trials and correct forced-choice trials share the same “Left” and “Right” states; “Right-NoRwd” and “Left-NoRwd” are the corresponding states for incorrect forced-choice trials. This is the true structure of the task as designed by the experimenters, as the same reward was available in forced-choice trials and free-choice trials if a correct choice was made. **(B) The six-state model**: each of the three odors leads to one of two states for left and right choices, with no generalization across odors. **(C) The hybrid-value model**: this model uses both the four-state and six-state representations (with a total of 10 states), with state values combined using weights *w*_4_ and (1 − *w*_4_) (illustrated as vertical boxes). **(D) The hybrid-learning model**: the same state representation and learning rule (green arrows, with learning rate *η*) as in the six-state model, with additional generalization (orange arrows, with generalization rate *η*_*g*_) between states representing valid forced choices and free choices. For simplicity, shown here only half of the learning and generalization updates (when reward is delivered in Left-Forced and Right-Free states), each representing generalization from forced-choice states to free-choice states or vice versa; the same rules apply to Right-Forced and Left-Free states. Boxes in white and gray represent rewarded and unrewarded states, respectively.

To examine the extent of generalization more directly, we constructed a series of reinforcement learning (RL) models with different state representations of the task (Fig 2), and tested how well these alternative models could predict the trial-by-trial choice behavior for each animal. In all models, animals learned the values of left and right actions for each odor through trial and error, choosing actions by comparing their values, with some decision noise (see Methods). What differed between the models was the assumed state representation, i.e., whether and how learning generalized across odors. The *four-state model* assumed full generalization between valid responses on forced-choice trials and corresponding responses on free-choice trials, with shared states between them; this model correctly reflects the generative structure of the task. The *six-state model* assumed no generalization between trial types, with separate states based on odor and action. We also considered two hybrid models to probe for partial generalization: the *hybrid-value model* combined values from the four-state and six-state representations at decision time, with a relative weight parameter *w*_4_. Finally, the *hybrid-learning model* assumed six states, but generalized across two pairs of states (“Left-Forced” and “Left-Free”; and similarly for right choices) with a generalization rate *η*_*g*_.

**Fig 3:**
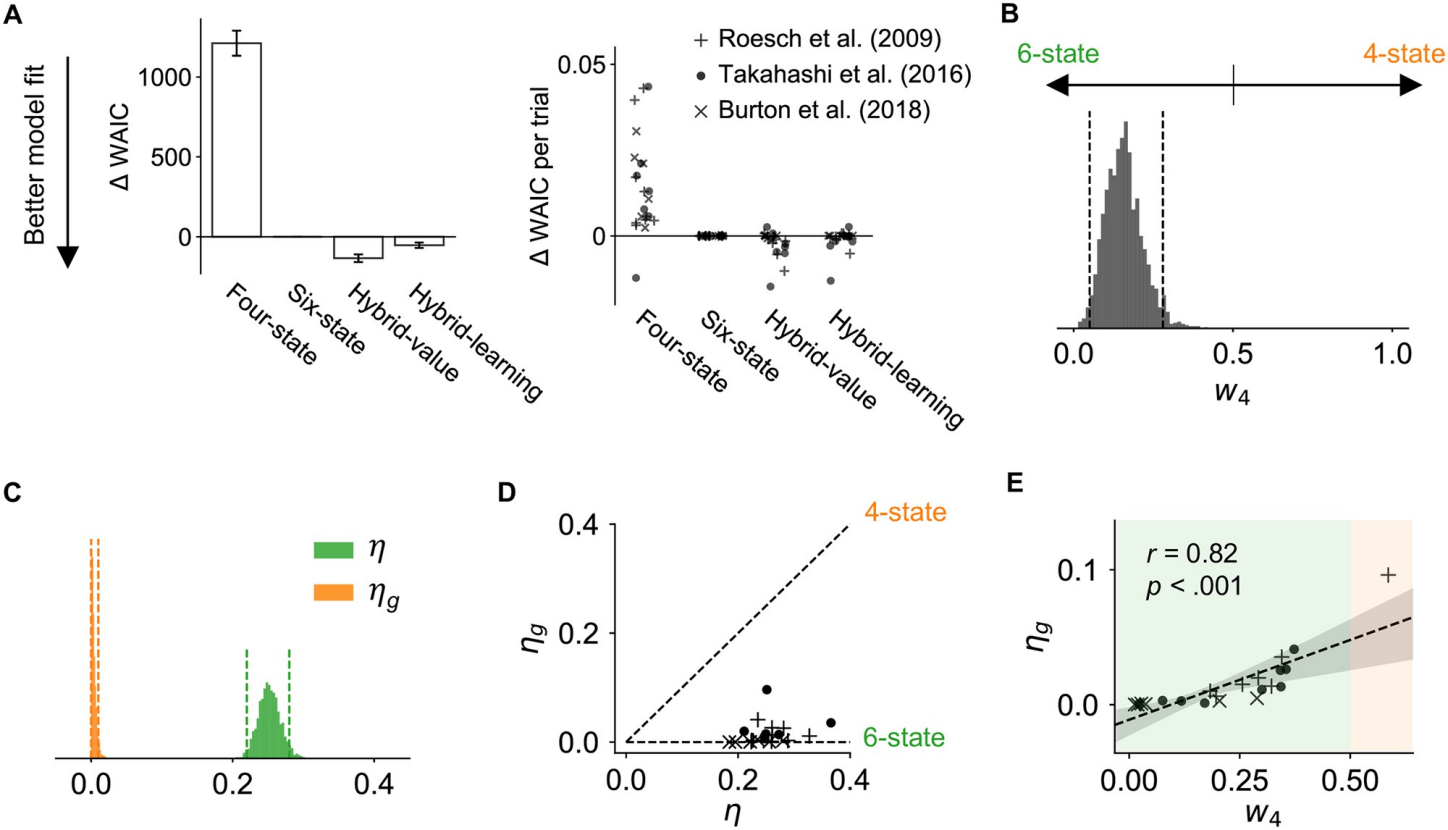
The six-state representation explains animals’ choices better than the four-state alternative. **(A)** Model comparison results. Left: WAIC difference between all four models and the six-state model for the entire dataset (summed across all trials from all animals). Lower values indicate better model fits. Error bars represent standard errors across samples [16]. Right: individual differences in average WAIC per trial between all models and the six-state model. Each marker corresponds to an individual animal, with different markers representing different datasets (same in D and E). **(B)** Posterior distribution of the group mean of four-state weight *w*_4_ in the hybrid-value model. Dashed lines represent 95% HDI. *w*_4_ = 0 corresponds to the six-state model, and *w*_4_ = 1 corresponds to the four-state model. **(C)** Posterior distribution of the group mean of learning rate *η* (in green) and generalization rate *η*_*g*_ (in orange) in the hybrid-learning model. Dashed lines represent 95% highest density interval (HDI). Generalization is almost negligible due to the low values of *η*_*g*_. **(D)** Posterior mean of *η* and *η*_*g*_ for each animal. The horizontal dashed line corresponds to the six-state model; the diagonal dashed line corresponds to the four-state model. **(E)** The correlation between *w*_4_ in the hybrid-value model and *η*_*g*_ in the hybrid-learning model at the individual level.

The free parameters of each model were fit to choice data from all animals using hierarchical Bayesian inference with Markov Chain Monte Carlo (MCMC) sampling [13, 14]. We evaluated model fits using the Watanabe–Akaike information criterion (WAIC; Fig 3A, lower values indicate better fits to data) [15]. Model comparison showed clear evidence for the six-state model, which out-performed the four-state model with a WAIC score that was 1211±78 (mean ± standard error across samples [16]) lower. The hybrid models were only slightly better than the six-state model (WAIC difference: −134 ± 24 for the hybrid-value model, and −53 ± 17 for the hybrid-learning model), suggesting little engagement of the four-state representation. Posterior estimates of the parameter values for the hybrid models revealed the dominance of the six-state representation: in the hybrid-value model, posterior estimates showed that the weight parameter *w*_4_ was smaller than 0.5 (equal reliance on the six- and four-state representations) both at the group level (95% HDI of group mean: [0.05, 0.28]; Fig 3B) and for all but one rat (Fig 3E). Similarly, in the hybrid-learning model, the learning rate *η* was an order of magnitude higher than the generalization rate *η*_*g*_ (95% HDI for group mean *η*: [0.22, 0.28], *η*_*g*_: [0, 0.01]; Fig 3C). In fact, most rats had a generalization rate close to zero (Fig 3D). These results indicate that, at the group level, rats did not acquire knowledge of the shared reward contingencies between valid forced-choice trials and free-choice trials, consistent with the earlier behavioral analysis. Instead, most rats appeared to learn about the two trial types separately.

Interestingly, we found marked heterogeneity in model fits at the individual level. Although for most animals the hybrid models did not predict choice better than the six-state model, for a subset of rats, model comparison indicated some degree of generalization (i.e., individual ΔWAIC of hybrid compared to the six-state model was negative; Fig 3 and S1 Fig). This indicated that the extent to which the animals recruited the four-state representation varied, which was confirmed by the span of individual parameter estimates of the generalization rate *η*_*g*_ and the four-state weight *w*_4_. Estimates of these two parameters were positively correlated at the individual level (*r* = 0.82, *p* < .001; Fig 3E), indicating the consistency of the two hybrid models. For only one animal, the four-state model fit better than the six-state model; this rat also had *w*_4_ > 0.5 and the largest *η*_*g*_ value. Comparing model fit for the first half to the second half of the behavioral sessions per individual showed the reliance on a shared representation was slightly stronger in later sessions (assessed both through comparison between the six-state and hybrid-value models, and the magnitude of the *w*_4_ parameter in the hybrid-value model; S2 Fig), suggesting generalization on this task may have emerged with experience.

**Fig 4:**
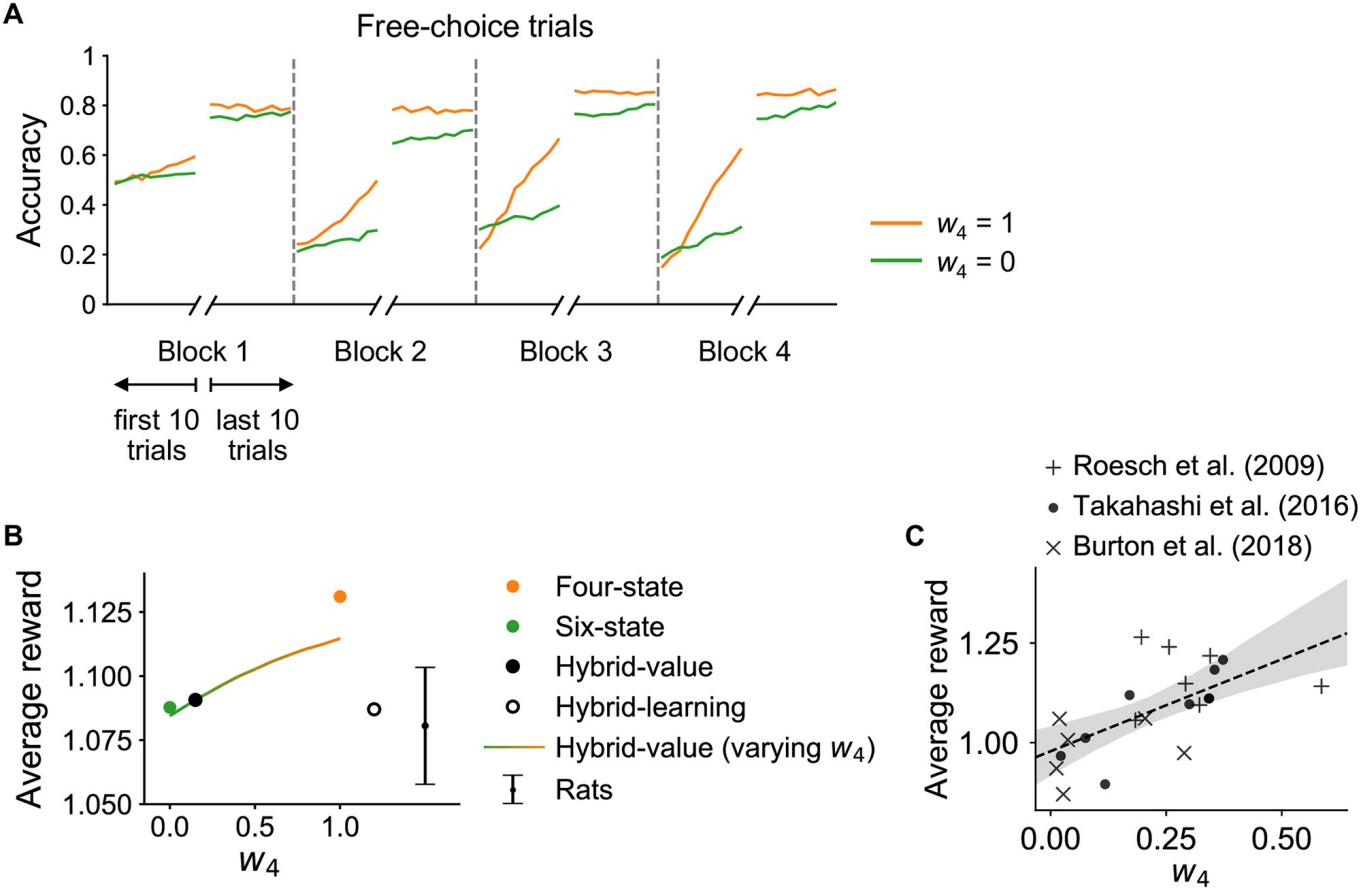
Simulations show faster learning, but a modest increase in reward earned, under the four-state representation. **(A)** Learning curves for free-choice trials in data simulated using the best-fit parameter values (i.e., posterior mean of the group-level parameters) of the hybrid-value model, but setting *w*_4_ to 1 or 0 (corresponding to the four-state and six-state models, respectively). The four-state model (*w*_4_ = 1) shows faster learning in each of the blocks and higher asymptotic accuracy than the six-state model (*w*_4_ = 0). **(B)** Average amount of reward per trial obtained by the models (dots and curve) and by animals (error bar, mean ± 1 s.e.m.). Dots represent model-simulation results obtained with their best-fit parameter values. The colored curve represents simulation results of the hybrid-value model with its best-fit parameters but varying *w*_4_ from 0 to 1. Average reward earned increases with *w*_4_, but the differences are relatively small (on the order of 5%). Rats performed, on average, in line with the six-state model and markedly worse than the four-state model. **(C)** Average reward obtained by each animal is positively correlated with their mean *w*_4_ parameter estimate (*r* = 0.64, *p* = 0.0015). Animals with a state representation more similar to four-state earned more reward on average. Dataset coded by marker type.

In principle, adopting a state representation that conforms to the true generative structure of the task should afford the most efficient learning and maximum accuracy, and thus maximize reward. To test the predicted performance of models that used different representations, we simulated choice behavior using the hybrid-value model with the best-fit group-level parameters (i.e., the “average rat”; see Methods). Changing the weight parameter w4 from 0 (equivalent to the six-state model) to 1 (equivalent to the four-state model) greatly accelerated learning in the early part of each block, as information could be appropriately generalized across trial types (Fig 4A). However, asymptotic accuracy at the end of a block was only slightly improved with shared reward representation, as was also reflected in the average reward yield in these simulations (Fig 4B). Indeed, despite the gains in learning after block changes, adopting a shared reward representation only increased reward per trial by ∼0.05 drops across the task. Thus, in this task at least, there was not strong pressure to learn a task representation that closely matches the generative structure of the environment.

## Discussion

Our results showed that most rats did not use a parsimonious state representation in the odor-guided choice task, even though, in principle, this representation could have helped them learn faster and earn more reward. Rather than exploiting a shared representation between valid forced-choice and free-choice trials, most rats learned the values of actions separately for each odor, with little to no generalization between trial types. This finding was consistent across both behavioral analyses and more detailed computational modeling approaches.

Why did most rats fail to exploit the shared reward structure of this task, even though rats have been shown to acquire quite complex task representations in other settings [17–19]? First and foremost, while the six-state representation is not as compact as the four-state and does not capture the true generative statistics of the task, it is sufficient to support good performance in this task: high accuracy for forced choice trials throughout the session and a reversal in preference between the left and right reward wells after block changes in the free choice trials. Indeed, our simulations found surprisingly little average benefit from learning the more compact four-state representation in terms of reward yield, suggesting that this task does not strongly incentivize acquiring such a representation. This might also imply that forming the more parsimonious task representation carries some cognitive cost. Indeed, the six-state representation assumes separable features for odor and location, while the four-state representation requires encoding the interaction between them. Accordingly, the prevalence of the simpler six-state representation in the choice behavior of these animals may be seen as less of a “failure” and more the result of a rational allocation of resources [20, 21].

Learned generalization in task representation has been shown in “acquired equivalence” [22], where animals respond equivalently to two stimuli that have been followed by the same consequence (e.g., food). If one stimulus is later paired with a new outcome (e.g., electric shock), the animals exhibit the same (fear) response to the other stimulus, demonstrating the shared representation. In the current task, however, odor cues only lead to the same consequence if followed by the correct action. The added complexity of instrumental contingencies perhaps limited the generalization strategies available in purely Pavlovian settings [23, 24]. The instrumental contingencies in this task may also prompt animals to use a different learning strategy entirely, acquiring a policy over available actions in a given state directly (e.g. [25]) rather than via action value representations as we have modeled here. In policy learning, generalization between odor trial types would be limited as alternative actions are grouped together, separating forced-choice from free-choice trials through the presence of the unrewarded choice option in these trial-types [26].

We found rats to segregate learning by trial type, with little generalization between them. This may indicate that odor representations dominated other features in this task. Rats display rapid learning, excellent memory and highly discriminative responses for odors, in line with the ethological relevance of these cues [27–29]. In contrast, a preference for spatial representation might have favored the four-state model. It may be that receiving reward at the well where choice was reported interfered with learning of spatial location as a dominant state component of the task. To better understand the conditions under which generalization may be acquired, it would be interesting to investigate other choice tasks that permit different representational strategies. For example, in a similar task in which reward identity (rather than delay) is changed between blocks [30], the distinct encoding of identities may help animals group together trials with the same reward identity, potentially facilitating generalization. Further, studying the very early stages of training, including the staggered introduction of different trial types, may demonstrate critical features of early experience that favor one type of task representation over another. Future work may also benefit from examining behavioral features beyond choices (e.g. reaction times), as well as neural representations, in order to further dissect the learning of task representation. Of note, we did not test for other forms of generalization across trials in our data, for instance, whether acquired knowledge about the block structure of the task facilitates faster learning after contingency changes in subsequent sessions. This kind of generalization has been observed in other odor-guided choice tasks as well as in numerous reversal paradigms [31, 32], and likely reflects learning of a higher-level structure of the task than we have investigated here.

Although none of the animals represented the generative task structure, few rats did acquire partial knowledge. This presents interesting directions for future studies: how was the partial knowledge acquired by these animals, and what gave rise to this individual difference in learning? Fitting our computational models to the first and second half of data separately provides some hints: overall, animals’ task representation was more hybrid towards the second half, and this was driven by a small subset of animals who relied more heavily on the shared representation in later sessions (S2(A)–S2(C) Fig). However, such representation learning did not result in a higher reward gain for these animals in later sessions (S2(D) Fig). It was unclear what contributed to the differential learning effects between animals as the amount of representational change was not associated with task experience (i.e., the number of sessions performed; S2(E) Fig). Nevertheless, we can conclude from our data that the acquisition of shared representations through experience is quite slow, and longer training experience may be needed to study this learning process.

Previous theoretical and empirical studies may help shed light on the principles of representation learning that facilitate generalization, as well as individual variability in this process. For instance, models of latent-cause inference propose animals use similarity to infer whether different experiences arise from a shared latent state [33–35]. Individual differences in task representations may also arise from idiosyncrasies in immediate experience, long-term effects of development or even genetic differences [36, 37].

In designing experiments, we often choose to randomize over irrelevant features, for instance, what side a stimulus is presented on, or whether an outcome is experienced through a forced- or free-choice trial (e.g., [38]). It is tempting to assume that our subjects also know to gloss over these nuisance task factors, however, learning to represent a task optimally is not a trivial process [39], especially when we cannot give subjects explicit instructions. Our results highlight that the factors influencing state representation in behaving animals extend beyond the experimenter-controlled generative statistics of a task, and reveal fine-grained differences in individual strategies that may be elicited in even a relatively simple reward learning task. Such discrepancies between the assumed representation and the one animals are actually using may be especially critical when interpreting neural data, but also in understanding behavioral data, and the effects of interventions. This suggests a humble approach to analysis that leans on the data—rather than an experimenter-centric view—to reveal how animals model the tasks they are performing.

## Methods

### Subjects

The behavioral data of 22 rats (322 sessions in total) performing an odor-guided choice task (see description below) were obtained from three previous studies [10–12]. Data from 7 rats (76 sessions) were obtained from [11]: these animals had electrodes implanted in their left ventral striatum for single-unit recordings (neural data not used in this paper; same for the other two studies). Data from 9 rats (75 sessions) were obtained from the control group in [10]: recording electrodes were implanted in their left or right ventral tegmental area. Finally, data from 6 rats (171 sessions) were obtained from the sucrose control group in [12]: self-administration catheters (that were used only for the cocaine group, not the control animals analyzed here) and driveable electrodes were implanted, and a twelve-day self-administration of maximum two sucrose pellets via lever press was completed a month before the experiment. All animals received extensive prior training on the task before data acquisition.

### The odor-guided choice task

#### Trial structure

Rats were trained on a well-studied odor-guided choice task [9]. The experiment apparatus is shown in Fig 1A. Each trial started with the illumination of a light inside the experimental box. When the light was on, a nose poke into the odor port resulted in the delivery of one of three distinct odor cues. At odor offset, the rat had 3 seconds to make a response at one of the two fluid wells located below and to the left or right of the odor port. One odor cue was reliably associated (through excessive pre-training) with reward delivery in the left well (a left forced-choice trial), a second odor was similarly associated with reward delivery in the right well (a right forced-choice trial), and a third odor was associated with reward delivery at either well (a free-choice trial). Odors were presented in a pseudorandom sequence such that 7 out of 20 trials were free choices, and the remaining were approximately equal numbers of left and right forced choices. If the rat made a correct response in a forced-choice trial, or either response in a free-choice trial, a reward was delivered, with a delay and a magnitude determined by the side of the well and the current block (see below for block structure); otherwise, the light would turn off immediately, signaling the end of the trial.

#### Block structure

Each session (one per day) consisted of four blocks (Fig 1B). All sessions started with two “delay blocks”, followed by two “magnitude blocks”. In “delay blocks”, reward (one drop of sucrose) at one well was delivered immediately (500ms; “short”), while reward at the other well was delayed (1–7s; “long”). The timing of the delayed reward varied according to an adaptive staircase procedure to ensure a fixed proportion of “long” free choices across individual rats (see respective papers from which data were reanalyzed for details). In “magnitude blocks”, the delay of reward was held constant (500ms), but the magnitude was one drop (“small”) at one well, and two drops in succession (“big”; drops 500ms apart) at the other well. Each drop of reward was a 0.05 ml bolus of 10% sucrose solution. For the first block of each session, the reward contingencies were assigned randomly to the two wells; they were then switched in the second block. In the third block, reward delivery at the “short” reward well remained the same (now called “small”) while delivery at the previously “long” well became “big”; these contingencies were switched again in the fourth block. All block switches were unsignaled. Blocks were on average 70 trials long, with varying lengths (standard deviation: 14 trials).

### Reinforcement-learning models with different state representations

We characterized the pattern of choices across a session using a series of reinforcement-learning (RL) models. We assumed the Rescorla-Wagner update rule [40], with reward discounted according to the delay between well entry and reward delivery, *d* (in units of seconds):

$$\begin{array}{c} V_{t+1}\left(s\right)=V_{t}\left(s\right)+\eta (\gamma^{d}r_{t}-V_{t}\left(s\right)), \end{array}$$ (1)

where *V*_*t*_(*s*) is the value of state *s* on trial *t* and *r*_*t*_ is the amount of reward (0, 1 or 2) delivered on the same trial. Learning rate *η* and discount rate *γ* were free parameters bounded in the range [0, 1].

We denoted the possible choices on each trial by *a* ∈ [left,right]. The decision variables governing the likelihood of left and right choices, *DV*(left) and *DV*(right), were determined by combining the value of the predicted state following that action (denoted by *s*_*a*_) with a side bias term *b* and a perseveration term *p*:

$$\begin{array}{c} DV\left(a\right)=V_{t}\left(s_{a}\right)+b\cdot I_{a,\text{right}}+p\cdot I_{a,a_{t-1}}, \end{array}$$ (2)

where $I_{i,j}$ is 1 for *i* = *j* and 0 otherwise. Thus, *b* < 0 indicates a general bias towards choosing the left side, and *b* > 0 indicates a bias towards right; *p* > 0 indicates a tendency to repeat the same choice as the previous trial (regardless of the odor cue), and *p* < 0 indicates a tendency to avoid the preceding choice and choose the alternate action.

Decision variables for the left and right choices were compared using a softmax (logistic) function to determine the probability of each choice, with a free parameter *β* controlling the randomness of choices (the slope of the logistic function). Finally, we also assumed a lapse rate of λ, where lapses involved a completely random choice.

$$\begin{array}{c} P\left(\text{left}\right)=\left(1-\lambda \right)\cdot \frac{1}{1+e^{-\beta (DV(\text{left})-DV(\text{right}))}}+\frac{\lambda }{2}. \end{array}$$ (3)

#### Alternative models

Core to all RL models is the state representation of the task with which an agent is engaged. For the current task, we considered two distinct representations: four-state and six-state. We built four alternative learning models: one each of the four-state and six-state representations, and two hybrid models with mixed state representations (Fig 2E).

- The *four-state model* assumed full knowledge of the shared reward representation. Thus, there were four subsequent states upon choice, with free-choice trials and correct forced-choice trials sharing the same subsequent states “Left” and “Right”. Reward outcomes in both trial types were used to update the value of these shared states. Incorrect forced choices led to two non-rewarding subsequent states “Left-NoRwd” and “Right-NoRwd”.
- The *six-state model* assumed no knowledge of the shared reward representation. Each odor led to a separate pair of subsequent left and right states (six states in total). Reward outcomes (including no reward upon incorrect choices) were used to update the value of the subsequent state determined by the current odor and choice.
- The *hybrid-value model* assumed both four-state and six-state representations, with two sets of state values updated in parallel following each outcome. When predicting choices, the hybrid model calculated the values for left and right choices using a weighted sum of the values under each representation:
  $$\begin{array}{c} V\left(s\right)=w_{4}V_{4}\left(s\right)+\left(1-w_{4}\right)V_{6}\left(s\right), \end{array}$$
  where *V*_4_ and *V*_6_ were the state values under four- and six-state representations, respectively, and *w*_4_ controlled the balance between the two representations. For *w*_4_ = 1, the hybrid-value model was equivalent to the four-state model, whereas for *w*_4_ = 0, it was equivalent to the six-state model, interpolating smoothly between the two models for the range of values of *w*_4_ ∈ [0, 1].
- The *hybrid-learning model* assumed a mixed representation. It had six subsequent states whose values were updated in the same way as in the six-state model, with learning rate *η*. In addition, generalization between free-choice trials and correct forced-choice trials occurred by using outcomes on those trials to update values of the other subsequent state with the same choice, with generalization rate *η*_*g*_. For *η*_*g*_ = 0, the hybrid-learning model was equivalent to the six-state model, whereas for *η*_*g*_ = *η*, it was equivalent to the four-state model, interpolating smoothly between the two models for the range of values of *eta*_*g*_ ∈ [0, *η*].

All four models had the following free parameters: *η*, *γ*, *β*, *b*, and *p*. In addition, the hybrid-value model had a free parameter *w*_4_, and the hybrid-learning model had a free parameter *η*_*g*_.

### Hierarchical model fitting using Stan

In order to test whether and to what extent rats acquired and took advantage of the shared reward structure of the task, we fit the above four RL models to their choice data. Hierarchical model fitting was performed with PyStan [41], where the parameters of individual animals are assumed to be drawn from a group-level distribution. For each model, we ran 4 chains of Hamiltonian Monte Carlo with 2000 iterations each (among which 1500 were warm-up samples). Model performance was evaluated using the Watanabe-Akaike information criterion (WAIC) [15], with a lower WAIC value indicating a better fit to the data. Results from this hierarchical fitting procedure were compared to those obtained by fitting each animal individually, and the parameter estimates and model comparison results were found to be consistent.

### Model simulation

Through hierarchical model fitting, we obtained posterior estimates of model parameters (both the group-level distribution, and individual parameters for each animal; S3 Fig). We then simulated the model to perform the task using these parameter values. The reward contingencies in the simulation matched the original experiment, including the block sequences, total number of trials per session, the proportion of forced-choice and free-choice trials, and the titration of the reward delay. For each model, we simulated a single agent governed by the group mean parameters (i.e., the “average rat”), which we used to calculate and compare the average amount of reward obtained (see Fig 4).

## Supporting information

S1 Fig Difference in WAIC per trial for each animal shows individual variability. We used the six-state model as a baseline to which we compared the four-state model (in orange), the hybrid-value model (gray) and the hybrid-learning model (white). Results are grouped according to the original study in which the data were first reported [10–12]. For the majority of animals, the four-state model fit much worse than the six-state. However, for a small subset, the four-state model performed equally well or even better (for rat 4) than the six-state model.
(TIF)

S2 Fig Split-half analysis shows slow learning of the shared representation through experience. **(A)** On average, the hybrid-value model provided only a modest improvement in model fit over the six-state representation in the first half of sessions per subject, however it showed a marked improvement in model fit over the six-state representation for the second half of sessions. **(B)** Most animals had very similar ΔWAIC (between hybrid-value model and six-state model; same below) in the first and second halves; a small subset had a lower ΔWAIC in the second half, representing an increase in use of the shared representation. Most animals had a higher *w*_4_ in the hybrid-value model in the second half of sessions than in the first half, also pointing towards greater generalization during the later sessions. **(C)** Difference in *w*_4_ between the second and first half of sessions was correlated with the difference in ΔWAIC between the second and first half of sessions. **(D)** Acquisition of the shared representation did not result in more reward gains: there was no correlation between either ΔWAIC or *w*_4_ difference (between the second and first half) with the reward amount difference (*p* = .69 and *p* = .21, respectively). **(E)** Having more task experience (more sessions performed) was not associated with a greater ΔWAIC or *w*_4_ difference (*p* = .16 and *p* = .74, respectively). Note the animals who had the largest changes in ΔWAIC magnitude experienced very few sessions. Two animals with only one session of data were excluded from this split-half analysis. Throughout: each dataset is coded by marker type indicating the original study [10–12].
(TIF)

S3 Fig Posterior estimates of parameter values in the hybrid-value model. From top to bottom: the group-level posterior distributions; the posterior means of individual parameters for each animal; MCMC samples of individual parameters (sampled from a distribution with the above mean and individual variances). Different colors indicate different animals; datasets are ordered according to the original study [10–12].
(TIF)

S4 Fig Learning curves for individual animals. Left: average accuracy over sessions; right: learning curves within a session (averaged across sessions), similar to Fig 1C but for each animal. Because of the noisiness of individual learning curves, only animals with over 10 sessions of data are shown. Animals are numbered in the same order as in Fig 1D and S1 Fig. These individual performance and learning curves largely resemble the group average.
(TIF)
