# Supplementary figures and images for: Minimal cross-trial generalization in learning the representation of an odor-guided choice task
Source: PLoS Comput Biol. 2022 Mar 25;18(3):e1009897. doi: 10.1371/journal.pcbi.1009897 (PMC8986096; doi:10.1371/journal.pcbi.1009897)

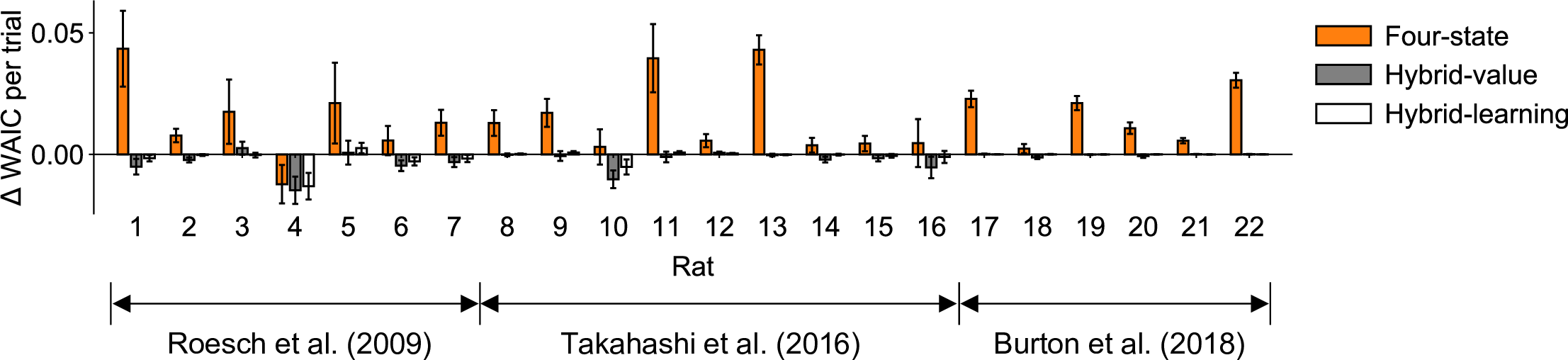

Supplement: S1 Fig — We used the six-state model as a baseline to which we compared the four-state model (in orange), the hybrid-value model (gray) and the hybrid-learning model (white). Results are grouped according to the original study in which the data were first reported [10–12]. For the majority of animals, the four-state model fit much worse than the six-state. However, for a small subset, the four-state model performed equally well or even better (for rat 4) than the six-state model. (TIF) [file pcbi.1009897.s001.tif]

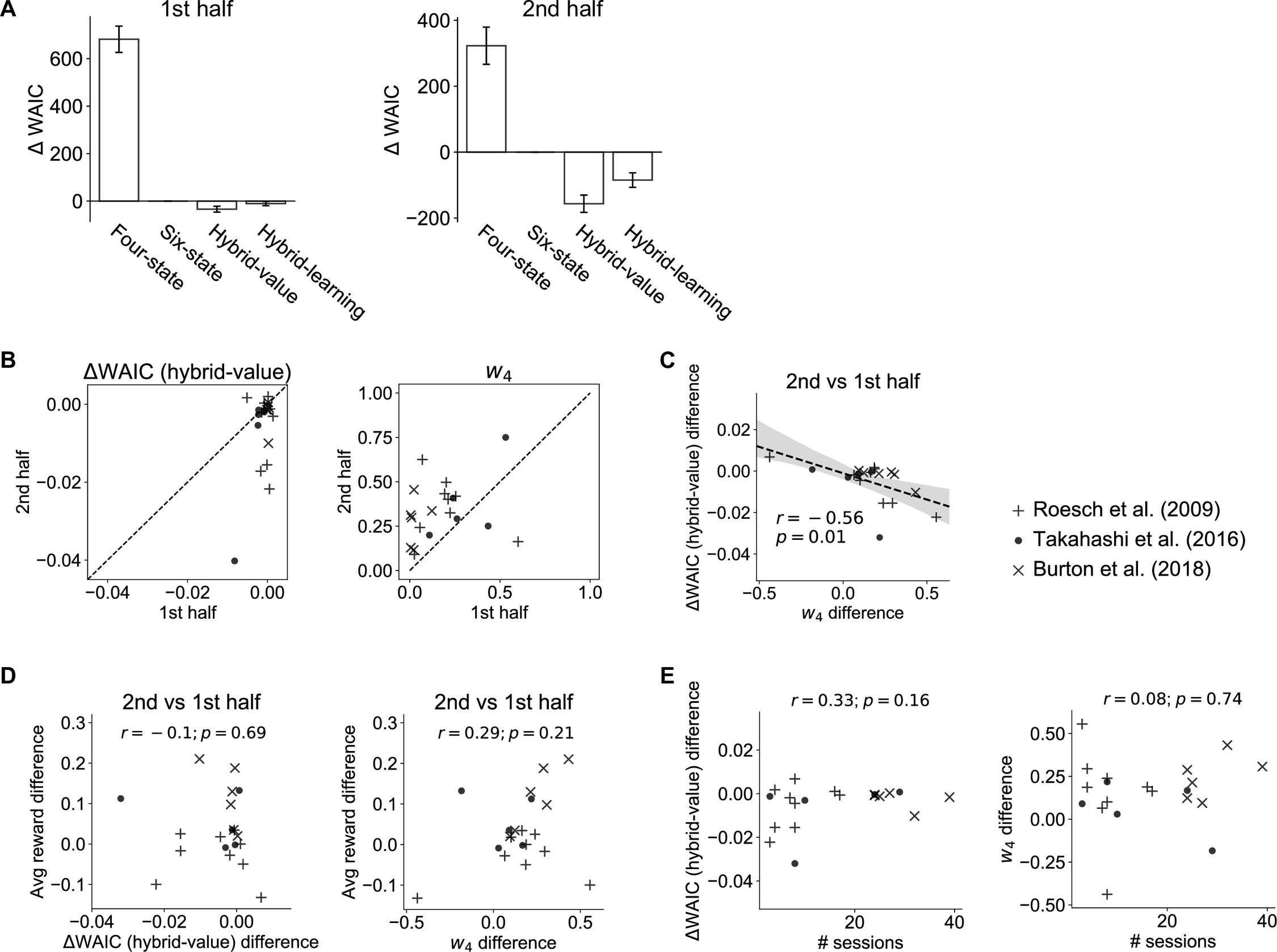

Supplement: S2 Fig — (A) On average, the hybrid-value model provided only a modest improvement in model fit over the six-state representation in the first half of sessions per subject, however it showed a marked improvement in model fit over the six-state representation for the second half of sessions. (B) Most animals had very similar ΔWAIC (between hybrid-value model and six-state model; same below) in the first and second halves; a small subset had a lower ΔWAIC in the second half, representing an increase in use of the shared representation. Most animals had a higher w4 in the hybrid-value model in the second half of sessions than in the first half, also pointing towards greater generalization during the later sessions. (C) Difference in w4 between the second and first half of sessions was correlated with the difference in ΔWAIC between the second and first half of sessions. (D) Acquisition of the shared representation did not result in more reward gains: there was no correlation between either ΔWAIC or w4 difference (between the second and first half) with the reward amount difference (p = .69 and p = .21, respectively). (E) Having more task experience (more sessions performed) was not associated with a greater ΔWAIC or w4 difference (p = .16 and p = .74, respectively). Note the animals who had the largest changes in ΔWAIC magnitude experienced very few sessions. Two animals with only one session of data were excluded from this split-half analysis. Throughout: each dataset is coded by marker type indicating the original study [10–12]. (TIF) [file pcbi.1009897.s002.tif]

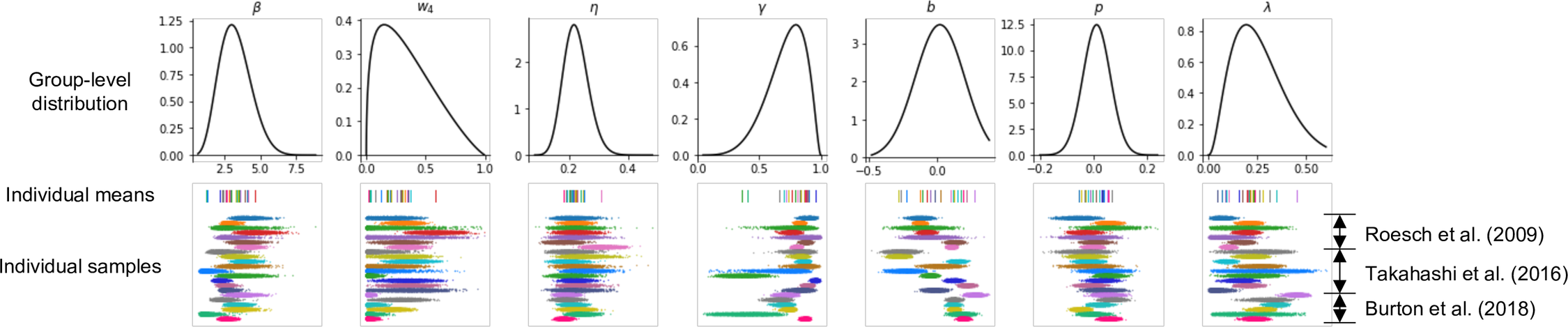

Supplement: S3 Fig — From top to bottom: the group-level posterior distributions; the posterior means of individual parameters for each animal; MCMC samples of individual parameters (sampled from a distribution with the above mean and individual variances). Different colors indicate different animals; datasets are ordered according to the original study [10–12]. (TIF) [file pcbi.1009897.s003.tif]

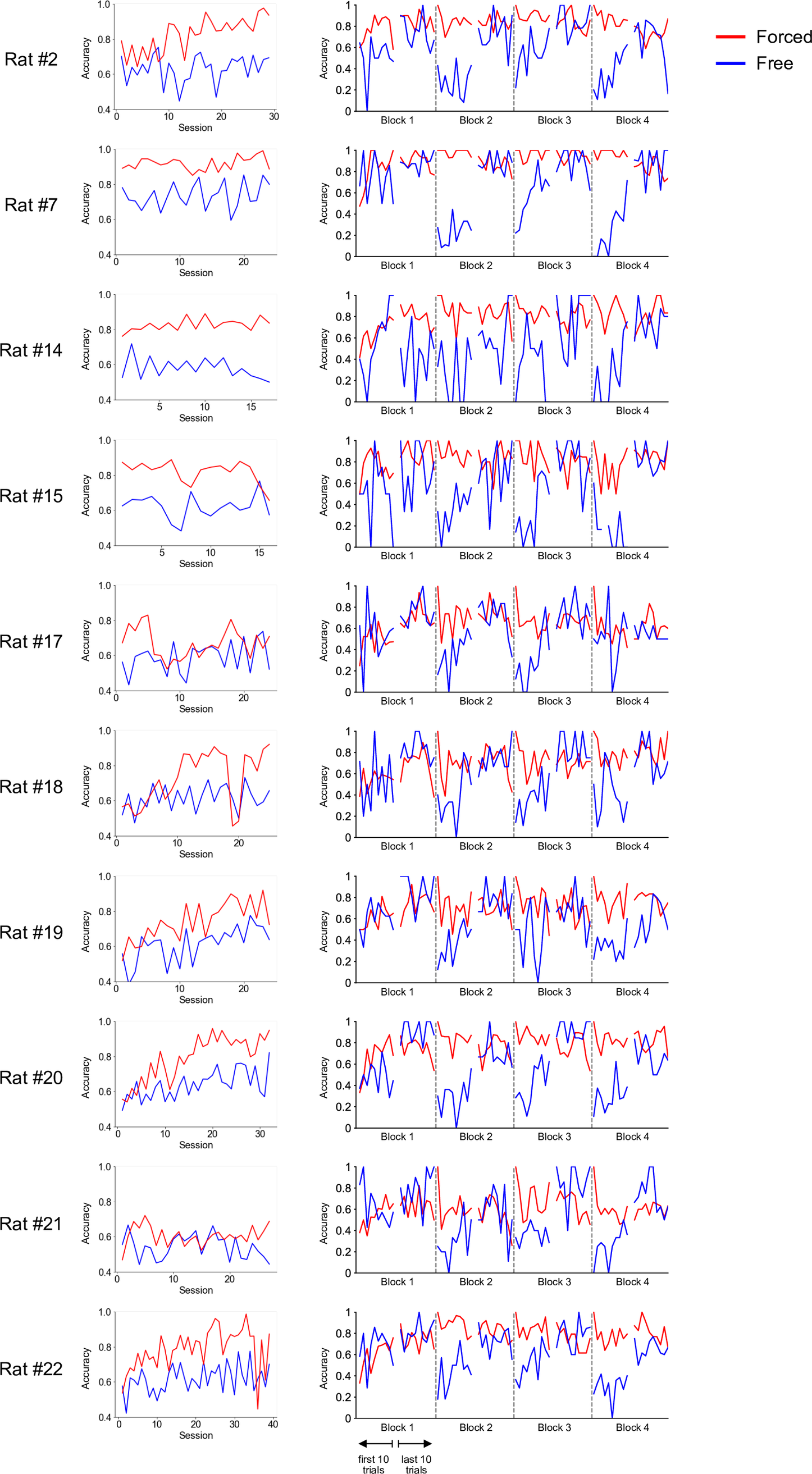

Supplement: S4 Fig — Left: average accuracy over sessions; right: learning curves within a session (averaged across sessions), similar to Fig 1C but for each animal. Because of the noisiness of individual learning curves, only animals with over 10 sessions of data are shown. Animals are numbered in the same order as in Fig 1D and S1 Fig. These individual performance and learning curves largely resemble the group average. (TIF) [file pcbi.1009897.s004.tif]
